# Primary Hepatic Neuroendocrine Tumor With Ectopic Adrenocorticotropic Hormone Production

**DOI:** 10.7759/cureus.22771

**Published:** 2022-03-02

**Authors:** Rajdeepsingh Waghela, Usman Ansari, Akshay Shetty, David Victor, Sudha Kodali

**Affiliations:** 1 Internal Medicine, Houston Methodist Hospital, Houston, USA; 2 Hepatology and Transplant Medicine, Houston Methodist Hospital, Houston, USA

**Keywords:** transarterial radioembolization, primary hepatic neuroendocrine tumor, liver transplantation, cushing's syndrome, ectopic acth production

## Abstract

This report describes the case of a 63-year-old female with a metastatic neuroendocrine tumor (NET). Imaging studies revealed a primary hepatic NET (PHNET) originating in the porta hepatis and associated with extensive hepatic metastasis. This represents an extremely rare presentation of PHNET associated with ectopic adrenocorticotropic hormone (ACTH) production and hypercortisolism. As such, it is a unique presentation of an otherwise rare pathology and hence we believe it contributes to the literature on PHNETs by supplementing it with information on an uncommon variation of an infrequent pathology.

## Introduction

Neuroendocrine tumors (NETs) arise from neuroendocrine cells, and often present with associated production of functionally active peptide proteins. These tumors most commonly originate in the gastrointestinal (GI) tract (55%) and the lungs (30%), although they may arise at any anatomical site. NETs comprise 1% of all GI tumors [1]. However, despite the liver being the most common site of metastasis of NETs, primary hepatic NETs (PHNETs) are rare entities. In fact, there have been less than 200 cases of PHNETs reported since 1958 when the first case was described, and they comprise only 0.3% of all NETs [2-3]. They are generally non-functional tumors, and only about 5% of patients present with the classic carcinoid syndrome findings of abdominal pain, diarrhea, and flushing [4]. Radiological diagnosis can be challenging, as PHNETs share many imaging features with primary or metastatic hepatic malignancies. While serotonin is usually the most common functional peptide produced by NETs, the patient discussed in this case had symptoms of hypercortisolism secondary to high levels of ectopic adrenocorticotropic hormone (ACTH) production from the PHNET. To our knowledge, there has been only one other case report of ectopic ACTH production by a PHNET in the literature so far [5].

## Case presentation

**Figure 1:**
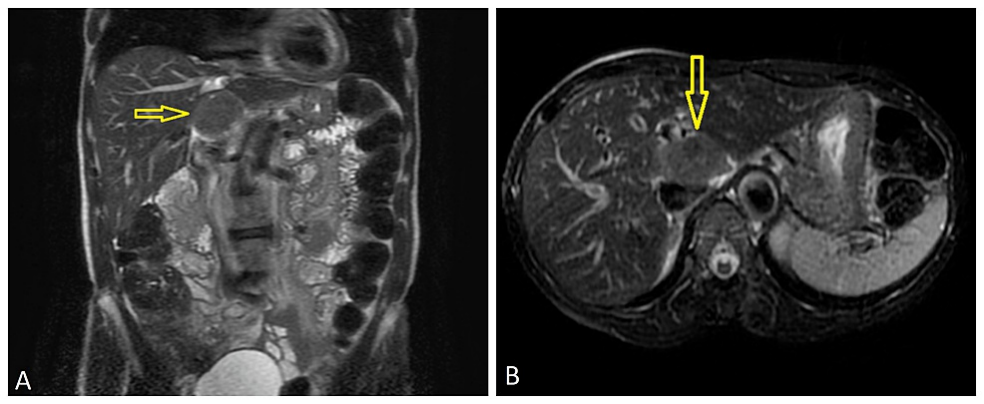
MRI abdomen with and without contrast A: Coronal view showing a 4.8 x 3.1-cm mass and numerous T1 hypointense lesions in right and left hepatic lobes. B: Axial view showing the same T2 isointense mass centered in the porta hepatis MRI: magnetic resonance imaging

The patient was a 63-year-old African American female with a past medical history significant for Sjogren’s syndrome and hypertension and surgical history of bowel resection secondary to small bowel obstruction in 1993. No further information could be obtained regarding the patient’s small bowel surgery and its indications. Therefore, while it was presumed that the patient’s NET was of a primary hepatic origin, there is a possibility that it may have been secondary to metastasis from a previously unknown small bowel NET from the 1990s. She initially presented with abdominal pain and reflux symptoms for which she underwent an esophagogastroduodenoscopy (EGD), which was negative, followed by abdominal ultrasound and MRI of the abdomen. The MRI was notable for numerous T1 hypointense masses throughout the right and left hepatic lobes. There was a large mass that was relatively T2 isointense on the liver centered in the porta hepatis measuring 4.8 x 3.1 cm; the lesions were noted to be indeterminate (Figure 1).

**Figure 2:**
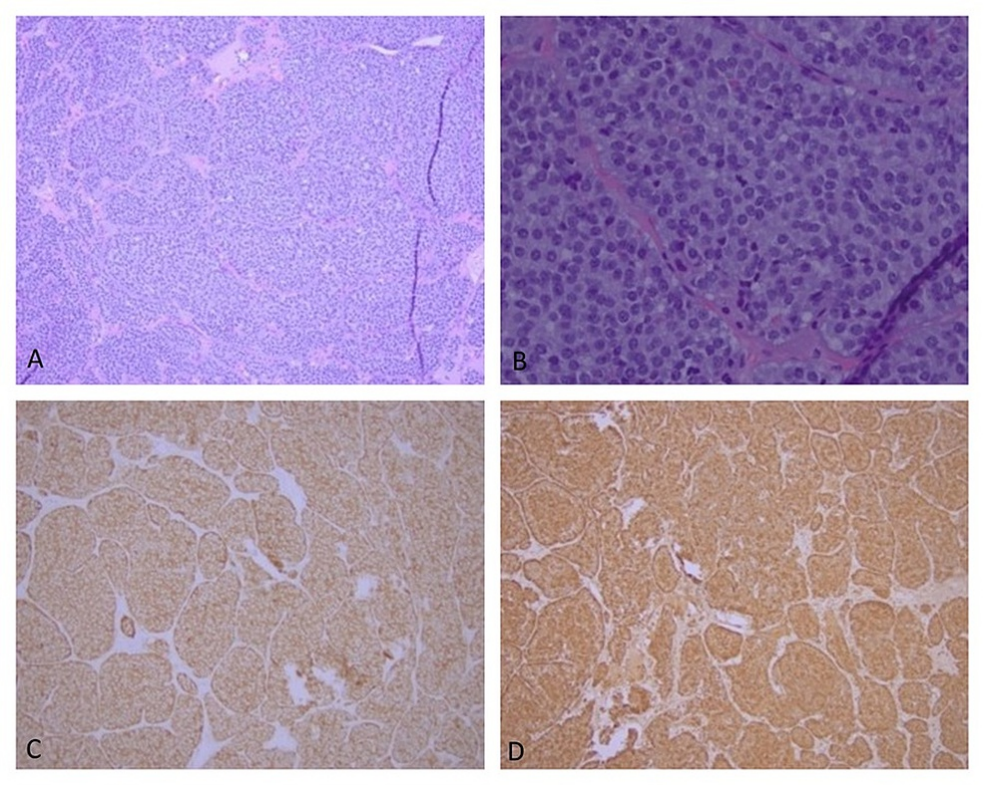
Immunohistopathology slides from liver mass biopsy A-B: Histopathological features of liver biopsy: sections show nests of tumor cells with a focal glandular pattern. Monotonous tumor cells are seen with finely granular chromatin. Mitoses are less than 2/2 mm^2^. MIB-1 (proliferation rate) is expressed in up to 15% of tumor cells (hematoxylin and eosin stain). C: Chromogranin positivity (immunohistochemistry stain). D: Synaptophysin positivity (immunohistochemistry stain)

While these masses were being evaluated, the patient presented with complaints of nausea and vomiting and was diagnosed with high-grade small bowel obstruction requiring hospitalization. The imaging findings were concerning for free air in the peritoneum, and during emergent exploratory laparotomy, there was no evidence of visceral perforation; however, a 2-cm superficial peripheral right liver lobe mass was noted and wedge-resection was performed. Immunohistopathology studies of the biopsy sample diagnosed NET of the liver. Immunohistochemical staining was positive for synaptophysin, chromogranin, and CD-56. Of note, ACTH staining of the tumor sample was negative (Figure 2).

Based on the above findings, the patient was diagnosed with a well-differentiated G2-grade NET. Serology at the time was notable for a serotonin level of 212 ng/ml (normal range: 50-220 ng/ml) and chromogranin A level of 9 ng/mL (normal range: 0-103 ng/ml).

**Figure 3:**
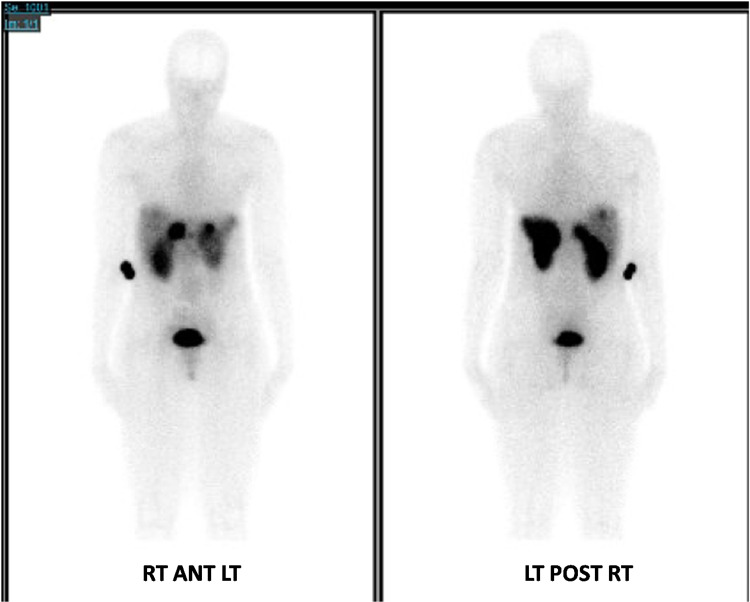
OctreoScan The images show multiple enhancing lesions in the liver with marked uptake near the porta hepatis. No extra-hepatic uptake was noted

An OctreoScan^ ^(Figure 3) one month later showed multiple enhancing lesions in the liver with increased uptake compatible with NET. There was marked uptake near the porta hepatis. Of note, there was no small-bowel, retroperitoneal, or mesenteric lymph node, or any extra-abdominal uptake seen.

A PET/CT NETSPOT Ga-68 dotatate scan one month later showed numerous bilateral lesions in the liver with a slight interval increase in the size and number of lesions. The previously observed marked porta hepatis uptake correlated with a caudate lobe metastatic mass. After a multidisciplinary tumor board discussion, diagnostic laparoscopy was recommended to determine her candidacy for right-liver lobe resection with simultaneous plans for evaluation for a liver transplant. The patient was subsequently started on monthly lanreotide infusions.

Diagnostic laparoscopy did not yield any evidence of extra-hepatic or lymph nodal disease. However, a large caudate mass was observed to be invading segments 2 and 3 of the liver, making resection an unviable option. Therefore, the patient was evaluated for a liver transplant as a potentially curative treatment.

Prior to definitive management, an extensive diagnostic evaluation was performed to exclude any metastasis or other sources of NET origin. MRI of the abdomen did not show any evidence of tumors or metastatic disease in the abdomen aside from primary hepatic tumors. CT of the chest did not show any suspicious findings. Endoscopic ultrasound (EUS) of the pancreas and retroperitoneal space did not yield any evidence of neoplastic disease. After undergoing liver transplant evaluation, the patient was approved for listing. She was closely followed up by the transplant team during this period. Her Model for End-stage Liver Disease (MELD) and sodium MELD (MELD-Na) score at the initial presentation was 6. Liver enzymes and tumor markers including beta-human chorionic gonadotropin (b-hCG) and carcinoembryonic antigen (CEA) were within the normal reference range. Of note, her alpha-fetoprotein (AFP) levels were elevated to 20.2 ng/ml at the initial evaluation.

While pending liver transplant, the patient was hospitalized for a hypertensive emergency with systolic blood pressure noted to be in the 200s at hospitalization. Her anti-hypertensive regimen was optimized, and the patient was discharged home. In the interim, she also underwent trans-arterial radioembolization (TARE) therapy with Yttrium-90 n for the caudate mass. Repeat MRI imaging of her abdomen after eight weeks was notable for an increase in the size and number of hepatic metastatic lesions, and there was also evidence of mild bilateral adrenal thickening.

Following TARE therapy initiation, the patient was once again hospitalized due to symptomatic hyperglycemia, during which her blood glucose was noted to be in the 400s, and hemoglobin A1c (HbA1c) was found to be 11%. Of note, her most recent HbA1c on labs from one year ago had been 6.2%. She was also found to be hypokalemic with potassium of 2.8 mEq/L.

Despite being discharged on an optimized insulin regimen, the patient was once again hospitalized one month later due to hypervolemia and anasarca. On examination, she was noted to have bilateral pre-tibial pitting edema and severe generalized myalgia, particularly worse in her proximal muscles. She was noted to be hypokalemic with potassium of 2.3 mEq/L on admission, and her bicarbonate level was 49 mEq/L. Given metabolic alkalosis, hypokalemia, acutely worsening hypertension, and new-onset diabetes mellitus, there was a concern for an adrenal etiology. The 24-hour urine electrolyte studies showed elevated urine potassium at 132.7 mmol/24 hours, decreased urinary creatinine at 793 mg/24 hours, and normal urinary sodium at 186 mmol/24 hours; 24-hour urinary free cortisol (UFC) was found to be significantly elevated at 5215.5 ug/24 hours. Serum AM ACTH level of 426.8 pg/ml and serum AM cortisol level of 68 ug/dl were observed. Serum aldosterone and renin were found to be within normal reference ranges.

Given the disproportionately elevated level of ACTH, the patient was suspected to have ectopic ACTH production likely originating from the NET. This prompted an MRI brain to exclude a pituitary source of ACTH production. It was unremarkable for any pituitary masses, unequivocally confirming likely ectopic ACTH production from a primary hepatic NET. The patient was started on both spironolactone 100 mg twice daily and triamterene 100 mg twice daily in addition to oral potassium chloride 20 mEq daily for the management of hypokalemia and hypervolemia on discharge. She was also started on ketoconazole 200 mg three times a day for the inhibition of steroidogenesis, with plans to add metyrapone if her symptoms did not improve.

The GI oncology team planned a re-evaluation of the tumor burden with a repeat NETSPOT scan on her as an outpatient to determine if the patient was a candidate for peptide receptor radionuclide therapy (PRRT) versus initiating systemic cytotoxic chemotherapy. However, the patient was unable to keep her NETSPOT scan appointment due to being re-hospitalized one month later for anasarca. She was started on an oral regimen of cytotoxic chemotherapy with capecitabine and temozolomide during that stay, with plans to continue therapy at home. The patient followed up with GI oncology as an outpatient after discharge. She refused chemotherapy and chose hospice care, and unfortunately passed away two months later.

## Discussion

Metastatic NET disease in the liver is common and is observed in 80% of cases at the time of diagnosis of the primary lesion. However, primary NETs of the liver are rare [2]. Neuroectodermal cells are the precursor cell type from which NETs originate. During the process of embryogenesis, neuroectodermal cells migrate from the neural crest throughout the body, with the notable exception of the liver where they are not routinely observed [6]. This mechanism may explain the rarity of PHNETs. Given that the PHNET precursor cells are not seen in the liver, theories postulating the pathogenic mechanism of the disease process often cite the presence of ectopic pancreatic or adrenal tissue as its source of origin [7]. An alternative theory suggests that PHNETs may arise from the argentaffin cells of the bile duct epithelium [8]. In both theories, the pathogenic mechanism involves chronic inflammation of the biliary system leading to intestinal metaplasia and consequently predisposing to the origination of PHNET. However, there is no definitive evidence for the pathophysiological mechanism as the origin of PHNETs.

Based on prior systematic reviews on PHNETs, their incidence is higher in women in their fourth and fifth decades of life [9]. NETs are further classified as functional or non-functional based on the presence of paraneoplastic manifestation arising from active hormone production. Carcinoid syndrome consists of a triad of flushing, diarrhea, and valvular heart disease and is classically associated with functionally active NETs due to ectopic serotonin production. NETs have been previously reported to secrete an assortment of functionally active hormones including chromogranin A, AFP, b-hCG, and CEA. However, PHNETs are generally classified as functionally inactive given the low amounts of hormone production, which are insufficient to manifest systemic effects [10]. Our patient was noted to have elevated levels of chromogranin A, as high as 1,561 ng/mL, and AFP levels as high as 23.6 ng/mL.

While there have been previous reports of functionally active PHNETs presenting with classical symptoms of carcinoid syndrome, there exists only one other report describing a functionally active PHNET with ectopic ACTH production to the best of our knowledge [1]. The patient reported in this case had obvious clinical signs of mineralocorticoid excess including multiple hypertensive emergencies and new-onset diabetes diagnosis. Her lab work was indicative of metabolic alkalosis and hypokalemia. She had an elevated serum ACTH level, and 24-hour urine studies showed a significant elevation in the UFC level. MRI of the abdomen was also notable for mild bilateral adrenal gland thickening. The high-dose dexamethasone test was deferred due to the aggressive course and rapid onset of symptoms, in association with elevated serum ACTH levels. Brain MRI showed a normal pituitary gland with no evidence of tumors, indicating that the PHNET was the obvious source of ectopic ACTH production.

Per prior reviews, the most common presenting complaint associated with PHNETs is abdominal pain followed by the incidental discovery of the mass in asymptomatic patients. Radiographic analysis of the mass with ultrasound, CT, or MRI has a low specificity for distinguishing PHNETs from other hepatic neoplasms. If a NET is suspected, OctreoScan using Indium-111-labeled octreotide is up to 90% sensitive and 83% specific. In addition to this, it has also been shown to detect an additional 16% of lesions missed by CT or MRI and is an important tool for preoperative evaluation and management planning [11].

Immunohistopathological diagnosis is the mainstay for the definitive diagnosis of PHNETs. The patient discussed in our case fits the demographic and epidemiological parameters associated with PHNET. One unique aspect of our case is that the patient’s PHNET was in fact functionally active; however, it was producing ectopic ACTH manifesting as Cushing’s syndrome. Primary surgical resection of the PHNET mass is the management of choice as it is associated with a five-year survival rate of around 80% [12]. However, even with negative resection margins, the recurrence rate is around 20% [5]. For patients in whom surgery is contraindicated, somatostatin analog therapy is an alternative treatment modality that can be pursued, as was done for our patient. Furthermore, other treatment options that have been studied in surgically unresectable PHNETs include TARE and trans-arterial chemoembolization (TACE) for targeted radio and chemotherapy. Radioactive agents such as Yttrium-90 or chemotherapeutic agents such as cisplatin can be added to further reduce tumor mass burden via cytoreduction [13].

Liver transplantation can be pursued in patients with unresectable disease and no evidence of metastasis. There are published guidelines for pursuing an adult MELD exception by the National Liver Review Board for liver transplantation in patients diagnosed with NET [14]. They stipulate that patients may qualify for a MELD exception in cases with neuroendocrine liver metastasis not amenable to resection if there is no recurrence within six months of extra-hepatic neoplastic disease resection. The criteria also stipulate that tumors should be of low-to-intermediate grade as per the WHO classification with a mitotic rate <20 per high power field (HPF) and less than 20% Ki-67 index positive markers. There are also imaging criteria involving either a triple-phase CT or MRI, which must be met to qualify for a MELD exception. A triple-phase CT scan must show a lesion on only one out of the three phases of the scan, with the exception that large lesions can become necrotic or calcified, and the arterial phase may demonstrate strong enhancement. MRI must characterize hepatic metastatic lesions as hypodense on T1 and hyper-vascular on T2 with diffusion restriction. The majority of lesions on MRI should be hyper-vascular on the arterial phase with wash-out during the portal venous phase [14].

The patient presented in this case met all criteria to qualify for a MELD exception for liver transplantation and was consequently referred for further evaluation prior to being approved for an orthotopic liver transplant. Additionally, in a case series by Fenwick et al. that followed up on patients with PHNET managed with liver transplantation, a significant recurrence-free survival of greater than three years was noted [12]. Therefore, liver transplantation is a theoretically curative treatment modality with improved recurrence-free survival and hence was offered to our patient. The role of systemic chemotherapy is generally limited in patients with unresectable disease as a palliative measure, and otherwise provides no survival benefit in patients with the surgically excisable disease. PRRT is another novel option being explored in NET patients with metastatic disease for improving survival rates. Kwekkeboom et al. found a 40-72 month survival benefit compared to controls in patients with metastatic NETs treated with PRRT using 177-Lutetium-DOTATOC [15]. Given the rarity of PHNETs, further evaluation is required to develop a standardized treatment regimen. However, for patients with extensive unresectable hepatic disease who may be eligible, liver transplantation should be considered.

## Conclusions

PHNETs are rare entities and may have a non-specific presentation and should be included in the differential diagnosis of any new liver lesions seen on imaging. PHNETs are rarely functional and may present with carcinoid syndrome-like symptoms. Our patient with PHNET had associated ectopic ACTH production manifesting as Cushing’s syndrome. Patients diagnosed with PHNET should be evaluated early in the course of their disease for the potential identification of rare functional PHNET manifestations such as ACTH secretion. While surgical resection should be offered to patients with resectable disease, liver transplantation is a treatment modality that should be considered in qualifying patients with unresectable disease.
